# Natural Polymers in Tissue Engineering and Regeneration: Material–Cell Mechanotransduction, Biofabrication Strategies, and Clinical Translation

**DOI:** 10.3390/biomedicines14040843

**Published:** 2026-04-08

**Authors:** Gabriela Calin, Mihnea Costescu, Marcela Nour, Camer Salim, Nicu Ovidiu Lungu, Alina Stefanache, Roman Rusnac, Elena Costescu, Mihai Cozmin, Petruta Iuliana Moraru, Alina Mitocaru, Tatiana Iov, Letiția Doina Duceac

**Affiliations:** 1 Doctoral School of Biomedical Sciences, “Dunărea de Jos” University of Galați, Research Centre in the Medical-Pharmaceutical Field, 47 Domnească Street, 800008 Galați, Romania; 2 Faculty of Medicine, “Apollonia” University of Iasi, Pacurari Str., 700399 Iasi, Romaniaiovtatiana@yahoo.com (T.I.); 3 Discipline of Pharmacology, Clinical Pharmacology and Pharmacotherapy, “Carol Davila” University of Medicine and Pharmacy, 020021 Bucharest, Romania; 4 Faculty of Medicine, “Ovidius” University of Constanța, 900601 Constanța, Romania; 5 Grigore T. Popa University of Medicine and Pharmacy, 700115 Iasi, Romania; alina.stefanache@umfiasi.ro; 6 Laboratory of Advanced Materials in Biopharmaceutics and Technics, Institute of Chemistry, Moldova State University, MD-2009 Chisinau, Moldova; 7 Faculty of Medicine and Pharmacy, “Dunărea de Jos” University of Galați, Research Centre in the Medical-Pharmaceutical Field, 800201 Galați, Romania; 8 Clinical Emergency Hospital for Children Sf. Ioan, 800487 Galați, Romania; 9 Institute of Forensic Medicine Iasi, Buna Vestire Str., No. 4, 700455 Iasi, Romania

**Keywords:** natural polymers, tissue regeneration, regenerative medicine, injectable polymers, 4D printing

## Abstract

Fractures are becoming a bigger and bigger global health problem, with an estimated 178 million new cases each year and 455 million people living with disabilities caused by fractures. Donor site morbidity, the risk of immune rejection, and limited functional integration all make current grafting techniques less effective. Biomaterials that come from nature, like collagen, gelatin, chitosan, alginate, hyaluronic acid (HA), and silk fibroin, have become promising scaffolds because they are bioactive, mimic the extracellular matrix (ECM), and can be broken down by enzymes. Crosslinking and composite reinforcement can greatly change how well they work. For example, collagen scaffolds that are highly crosslinked with glutaraldehyde keep up to 51.9% of their tensile strength after being exposed to enzymes, while non-crosslinked scaffolds only keep 12% of their strength. Chitosan–hydroxyapatite matrices, on the other hand, can reach compressive strengths of 2–12 MPa, which is close to the strength of cancellous bone. Additive manufacturing and 4D printing allow for precise control of structures and the ability to change their shape over time, which helps with vascularization and mechanical adaptation. Injectable and in situ-forming hydrogels show clinically important results, such as filling 85% of osteochondral defects in rabbits, improving left ventricular ejection fraction by up to 9% in large-animal cardiac models, and speeding up healing by 25–40% in chronic wounds. Even with these improvements, it is still hard to get batch consistency, a standardized way to test mechanical properties, and production that meets GMP (Good Manufacturing Practices) standards and can be scaled up.

## 1. Introduction

Fractures and soft tissue injuries (heart tissue, peripheral nerves and skeletal muscle) are a huge and growing problem for global health. This is because populations are getting older, osteoporosis and chronic metabolic diseases are becoming more common, and trauma is always a risk. There were about 178 million new fractures around the world in 2019 (with a 95% uncertainty interval of 162–196 million). This is a 33.4% increase since 1990. That year, 455 million people were living with the short- or long-term effects of fractures. This was a shocking 70.1% rise over the previous three decades, which meant that 25.8 million years were lived with disability (YLDs)—a 65% rise since 1990. In 2019, there were 2296.2 fractures for every 100,000 people, with the highest rate being 15,381 cases for every 100,000 people aged 95 and older. There are at least 37 million fragility fractures each year, mostly in people over 55. That is about 70 fractures every minute around the world. These numbers show that there is an unstoppable and growing epidemic of bone-related disabilities and health-care costs [1,2,3,4,5].

Current clinical solutions, such as autografts, allografts, and synthetic implants, have some serious problems. Autografts, frequently regarded as the gold standard, are limited by donor site morbidity and restricted availability; allografts pose risks of immune rejection and disease transmission; synthetic biomaterials often do not achieve complete integration or predictable degradation. These problems show that there is a growing need for regenerative therapies that not only replace tissue but also restore its structure and function. Tissue engineering is a promising solution that aims to repair damaged tissues by combining scaffolds, cells, and signaling factors. But designing the perfect scaffolds is still hard. They need to provide enough mechanical support, let cells attach and differentiate, break down in a controlled way as new tissue forms, and support the integration of blood vessels and nerves [6,7,8,9].

In this context, naturally derived polymers like collagen, gelatin, hyaluronic acid (HA), and chitosan have a lot of benefits. These materials imitate the native extracellular matrix (ECM), presenting established biochemical motifs: collagen’s integrin-binding domains, HA’s CD44-mediated signaling, and chitosan’s antimicrobial and hemostatic characteristics, delivering both structural and bioactive support for regeneration [10,11]. They are biodegradable, metabolized into non-toxic byproducts, enabling dynamic tissue remodeling [12]. Modern technologies like electrospinning, freeze-drying, and 3D/4D bioprinting have greatly increased their ability to be made into hydrogels, sponges, fibers, and injectable formulations, giving precise control over their mechanical properties, porosity, and architecture [13,14].

Quantitative data on degradation rates and mechanics further demonstrate the advantages and difficulties associated with these materials. Collagen scaffolds that do not crosslink can break down quickly, losing about 33.5% of their mass in just 96 h and 88% of their ultimate tensile strength when enzymes break them down. On the other hand, scaffolds that were highly crosslinked with 2.5% glutaraldehyde for 1 h only lost 1.9% of their mass and kept 51.9% of their tensile strength during the same time [15]. The degradation of silk fibroin scaffolds can also be controlled: scaffolds made from water lost 90% of their mass after 24 h of exposure to protease XIV, while scaffolds made with HFIP (hexafluoroisopropanol) solvent kept 70% of their mass even after 21 days [16]. Membranes that come from nature usually break down in vitro within 7 to 10 days unless they are crosslinked. On the other hand, composites or crosslinked versions break down much more slowly, providing more structural support [17].

Recent developments in mechanobiology have demonstrated that cells are active sensors of their physical surroundings rather than just passive inhabitants of a scaffold. Through intricate intracellular signaling pathways like Yes-associated protein (YAP) and transcriptional coactivator with PDZ (PSD95, Dlg and ZO-1)-binding motif (TAZ) translocation, the mechanical stiffness of a substrate can determine stem cell lineage specification, guiding mesenchymal stem cells (MSCs) toward osteogenic lineages on stiff matrices or neurogenic lineages on soft matrices. In order to maximize these mechanotransductive signals, the design of natural polymer scaffolds has changed to concentrate on the exact control of crosslinking density, viscoelasticity, and degradation kinetics. Natural polymers’ “instructional” ability enables biological responses to be programmed, going beyond basic biocompatibility to bioactive regeneration [18,19,20].

## 2. Natural Polymers and Composites

In recent years, the use of naturally derived polymers in tissue engineering has come a long way. This is because there is a need for bioactive, biocompatible materials that can support the complex chain of cellular events that happen during tissue repair. Their structural and biochemical similarity to the native ECM, along with their capacity to degrade into non-toxic byproducts, renders them appealing substitutes for entirely synthetic polymers. These benefits are especially important in orthopedic and reconstructive settings, where successful outcomes depend on integration with host tissue, vascularization, and long-term stability [10,21,22,23,24].

Physical treatments, such as dehydrothermal (DHT) treatment and UV irradiation, eliminate harmful chemical residues while producing reduced crosslinking densities and stiffnesses. Under moderate physiological settings, enzymatic techniques such as transglutaminase or horseradish peroxidase (HRP) promote the formation of particular bonds (e.g., isopeptide bonds between glutamine and lysine). These approaches are very useful for generating hydrogels in situ while minimizing cytotoxicity [25,26,27,28].

### 2.1. Natural Polymers in the Biological Environment (ECM-Mimicking, Enzymatic Degradation)

The shift from bioinert to bioactive materials in regenerative medicine is fundamentally driven by the use of naturally derived biomaterials that uniquely recapitulate the structural and functional complexity of the native extracellular matrix (ECM) [29,30,31]. These materials provide an evolutionary reservoir of biochemical signals, most notably the Arg-Gly-Asp (RGD) sequence found in proteins like collagen and fibrin, which directly engages cell-surface integrins to activate intracellular signaling cascades that regulate survival, proliferation, and lineage commitment. Unlike synthetic polymers that often lack cell-recognition motifs, natural substrates such as the small intestinal submucosa (SIS) and bladder submucosa (BS) are inherently rich in glycosaminoglycans (GAGs), fibronectin, and vitronectin, providing a tissue-specific niche that facilitates host integration and vascularization [32,33,34,35].

The cell compatibility of these materials is further enhanced by their ability to provide the necessary microarchitecture for deep cellular infiltration and functional attachment. For instance, silk fibroin (SF) enhanced with chondroitin sulfate or hyaluronate has been shown to improve chondrocyte biosynthesis and matrix accumulation, effectively mimicking the cartilaginous environment [36,37]. Similarly, fibrin gels serve as a provisional matrix during hemostasis, supporting rapid cell migration and inducing the differentiation of stem cells into specialized lineages, such as insulin-producing cells or myocardial fibers, depending on the localized mechanical and chemical cues [38,39,40].

Enzymatic degradability is a critical physiological advantage of natural polymers, enabling the synchronized transition from scaffold resorption to the deposition of native host tissue, a process known as constructive remodeling. Fibrillar collagens (Types I, II, and III) are highly resistant to most general proteases but are specifically cleaved at a helical locus—typically 3/4 from the N-terminus—by matrix metalloproteinases (MMPs) such as MMP-1, MMP-8, and MMP-13. These enzymes unwind the triple helix into gelatin, which is then further degraded by gelatinases like MMP-2 and MMP-9 into soluble fragments [41,42,43]. Chitosan degradation is primarily mediated by lysozyme, a glycoside hydrolase that cleaves the β-1,4-glycosidic linkages between N-acetylmuramic acid and N-acetyl-D-glucosamine. The degradation rate can be precisely tuned by modifying the degree of deacetylation (DD), as lysozyme specifically targets acetylated units [44,45,46]. Fibrin is resorbed via plasmin-mediated fibrinolysis, where tissue plasminogen activator (tPA) converts plasminogen to the serine protease plasmin, which then hydrolyzes the fibrin network into soluble degradation products (FDPs) [47,48,49,50].

Mechanobiology focuses on how physical cues like stiffness and geometry regulate cellular behavior through mechanosensing mechanisms. Central to this process is the transcriptional coactivator yes-associated protein (YAP) and its homolog, transcriptional coactivator with PDZ-binding motif (TAZ). On stiff substrates, YAP/TAZ are dephosphorylated and translocate into the nucleus, where they bind to TEAD transcription factors to drive proliferation and osteogenic differentiation. On soft substrates (<2 kPa), YAP/TAZ are sequestered in the cytoplasm and undergo degradation, favoring adipogenic or chondrogenic fates. Recent studies have identified subnuclear adhesions as rapid mechanotransduction nodes that cause YAP/TAZ relocalization within minutes of a mechanical transition [51,52,53,54].

The expansion of tissue engineering materials includes both refined synthetic polyesters and novel bioactive molecules such as polysialic acid (PSA) and self-assembling peptides (SAPs). Saturated poly(α-hydroxy esters) like PLA, PGA, and PLGA remain dominant due to their controllable degradation via hydrolytic de-esterification. PLGA 50:50 exhibits the fastest degradation (weeks), while ratios like 85:15 allow for slow release over 3–6 + months. However, their acidic byproducts can trigger inflammation, necessitating hybridization with natural polymers like collagen or chitosan to buffer the environment and improve cell adhesion [55,56,57,58].

Polysialic acid (PSA) is a unique homoglycan that mimics the embryonic glycocalyx and modulates neural progenitor cell (NPC) plasticity and migration. PSA is non-immunogenic and can be specifically degraded by phage-born endosialidases or endogenous neuraminidases. In stroke models, PSA-functionalized scaffolds reduce neuroinflammation and promote neural tissue regeneration by interacting with inhibitory Siglec receptors on macrophages and microglia [59,60,61,62,63]. Self-assembling peptides (SAPs), such as RADA16, spontaneously organize into β-sheet nanofibers in response to physiological pH and ionic strength, creating a nanofibrous mesh that resembles native ECM and supports axonal growth. While SAP networks are generally weak, their mechanical properties can be significantly enhanced through covalent crosslinking with agents like genipin [64,65,66,67].

### 2.2. Natural Polymers and Composites in Tissue Engineering

Collagen, the most common structural protein in mammals, makes up about 25–30% of all the protein in the body and up to 90% of the organic matrix of bone. Collagen type I scaffolds have been demonstrated to enhance cell viability (>90% after 7 days in vitro) and osteogenic differentiation in tissue engineering, especially when crosslinked to augment mechanical performance. Freeze-dried collagen scaffolds can have porosities of 70% to 95%, which make it easier for nutrients to move through them and blood vessels to grow. But non-crosslinked collagen usually breaks down in vivo in 4–6 weeks, which can cause the mechanical integrity to break down too soon before the tissue is fully remodeled [68,69,70,71]. Xenogeneic collagen derived from bovine or porcine sources may elicit immunogenic reactions unless extensively purified, limiting its use in certain patient populations. Most clinical collagen is xenogeneic (bovine or porcine). While telopeptide removal (atocollagen) reduces immunogenicity, it does not eliminate the risk of zoonotic disease transmission (e.g., BSE) or alpha-gal allergic responses. This has necessitated rigorous, often harsh, purification protocols that can denature the protein, reducing its bioactivity [72,73,74,75,76].

GA enhances stiffness and slows breakdown by bridging amine groups in proteins like collagen. For example, collagen scaffolds that are heavily crosslinked with GA maintain up to 51.9% of their tensile strength following enzymatic treatment, compared to just 12% for non-crosslinked versions. The release of leftover aldehydes during decomposition can cause cytotoxicity and calcification, which limits their use in non-calcified tissue engineering applications such as cartilage or nerve regeneration [77,78].

Gelatin, derived from the partial hydrolysis of collagen, preserves numerous cell adhesion sequences, including Arg-Gly-Asp (RGD) motifs, which facilitate integrin-mediated cell attachment. When made without crosslinkers, gelatin hydrogels usually have compressive moduli between 5 and 30 kPa. When chemically modified with agents like genipin or glutaraldehyde (GA), they can reach 50 to 100 kPa [79,80,81,82]. These hydrogels break down faster than collagen, usually within 2 to 3 weeks in vivo. This makes them good for uses where the scaffold needs to be changed quickly, like wound dressings or short-term growth factor delivery. But because they are not very strong, they cannot be used in load-bearing tissues unless they are mixed with stiffer polymers or ceramics [83,84,85,86,87].

Genipin is a natural crosslinker produced from *Gardenia jasminoides* that has a reduced cytotoxicity profile (approx. 5000–10,000 times less toxic than GA), but it provides equivalent mechanical reinforcement [88]. Genipin works by bridging primary amine groups (for example, lysine in collagen or chitosan), which typically results in a distinctive blue coloration. According to studies, genipin-crosslinked collagen gels can control stiffness between 0.029 kPa and 12.5 kPa, which is adequate to guide MSC development from visceral smooth muscle to vascular smooth muscle lineages [89,90]. The crosslinking process comprises a nucleophilic assault by primary amines on the ester group of genipin, followed by a polymerization event that produces long-range crosslinks, as opposed to the short-range “zero-length” crosslinks created by 1-ethyl-3-(3-dimethylaminopropyl) carbodiimide (EDC) combined with N-hydroxysuccinimide (NHS) that facilitate the formation of covalent bonds between polymer chains [91].

Chitosan is a cationic polysaccharide obtained from the deacetylation of chitin that has been thoroughly investigated for its intrinsic antibacterial properties, which result from the disruption of bacterial membranes through electrostatic interactions. Chitosan-based scaffolds mixed with hydroxyapatite can reach compressive strengths of 2 to 12 MPa, which are similar to that of cancellous bone [92,93,94,95]. They have also been shown to support mineral deposition in vitro [96]. Chitosan breaks down mostly through hydrolysis mediated by lysozyme. The time it takes to break down varies from a few weeks to a few months, depending on how much deacetylation and molecular weight it has. But because it does not dissolve well at physiological pH, it needs to be chemically changed, for instance, by quaternization, to make it easier to work with and more useful for biological purposes [97,98,99].

When cells attach to a stiff substrate (e.g., a strongly crosslinked collagen or chitosan–hydroxyapatite scaffold, E > 30 kPa), integrin clustering forms persistent focal adhesions and assembles a highly tensioned actomyosin cytoskeleton. This cytoskeletal strain physically deforms the nucleus, causing nuclear pores to open or transport protein shape to change, thereby increasing YAP/TAZ nuclear translocation. Once in the nucleus, YAP/TAZ bind to TEAD transcription factors, upregulating genes related to proliferation and osteogenesis, such as Runx2 and Col1a1 [100]. This is why mineralized collagen scaffolds, which are stiffer, promote stronger bone development than soft hydrogels. Soft substrates (e.g., mildly crosslinked alginate or hyaluronan hydrogels, E < 1 kPa) do not allow cells to create strong traction forces. The cytoskeleton stays relaxed, but the Hippo signaling pathway kinases (LATS1/2) are active. These kinases phosphorylate YAP/TAZ, indicating that they will be retained in the cytoplasm and degraded by proteasomes. This condition limits proliferation while promoting adipogenic or neurogenic differentiation pathways [101].

Alginate, which comes from brown seaweed, gels quickly when divalent cations like Ca^2+^ are present. This makes it a good choice for bioink for 3D bioprinting that uses extrusion. Pure alginate hydrogels have compressive moduli that are not very high (<1 MPa) and do not have native cell-binding sites. RGD-functionalized alginate can greatly improve cell adhesion and growth [11,102,103,104]. In vivo, alginate degradation transpires over a duration of weeks to months, influenced by crosslinking density and ion exchange with physiological fluids. Alginate’s controllable degradation profile makes it good for uses like cartilage repair, where it is important for the scaffold to slowly break down [105,106].

Silk fibroin is obtained from *Bombyx mori* cocoons and possesses a β-sheet-rich crystalline structure that provides remarkable tensile strength, with reported values reaching 740 MPa and elongation at break up to 15% [10,107,108]. This material is strong and does not break down quickly due to enzyme action (in vivo, it can take more than 12 months), so it can be used to make ligaments and tendons, as well as load-bearing bone scaffolds. However, unmodified silk fibroin is somewhat hydrophobic, which can make it harder for cells to stick to it at first. To make it more bioactive, peptides or plasma treatments are often used to change the surface [109,110,111,112].

HA is a linear glycosaminoglycan found in connective, epithelial, and neural tissues, and it is especially valued for its ability to control inflammation and encourage the growth of new blood vessels. In vivo, native HA has a very short half-life, usually less than 24 h, because hyaluronidases break it down quickly [113,114,115]. To enhance its functionality, HA is often chemically crosslinked (for instance, with divinyl sulfone or methacrylate groups), resulting in hydrogels with degradation times varying from days to several weeks. These systems have demonstrated potential in cartilage regeneration and soft tissue repair, with clinical studies indicating enhanced histological scores relative to untreated controls [116,117,118].

Fibrin is a naturally occurring biopolymer generated during the coagulation cascade, making it a simple and non-toxic option for autologous scaffolding [18]. Unlike synthetic polymers, fibrin functions as a temporary matrix that the body is designed to reconstruct. Fibrin gels are generated from the enzymatic breakdown of fibrinogen by the serine protease thrombin. This process liberates fibrinopeptides A and B, revealing polymerization sites that allow fibrin monomers to self-assemble into a fibrous network. The structure is distinguished by a nanofibrous architecture that resembles the natural temporary ECM observed in wound beds [119]. Pure fibrin hydrogels are intrinsically soft and mechanically weak, with tensile strengths generally less than 0.01 MPa and Young’s moduli ranging from 1 to 10 kPa, depending on the fibrinogen and thrombin concentrations utilized during polymerization. While fibrin’s low stiffness resembles soft tissues such as the brain or marrow, it is limited in its usefulness in load-bearing scenarios unless supplemented by synthetic polymers (e.g., PCL) or chemical crosslinkers such as genipin [120,121].

Fibrin is peculiar in that it degrades quickly and enzymatically. It is digested by plasmin (which is produced from plasminogen by tissue plasminogen activator) in a process known as fibrinolysis. In vivo, unaltered fibrin scaffolds can disintegrate in days to two weeks. While quick wound healing is beneficial, it is frequently too fast for tissue regeneration. The use of protease inhibitors such as aprotinin or tranexamic acid enables researchers to adjust the breakdown rate to match the rate of neo-tissue development [122].

Agarose is a neutral polysaccharide extracted from marine red algae and is distinguished by its unique thermoreversible gelation and mechanical robustness, characteristics that have made it a staple in cartilage and bone tissue engineering. The material forms hydrogels through physical hydrogen bonding upon cooling; while the polymer chains exist as random coils in solution at high temperatures, they undergo a coil-to-helix transition upon cooling (typically below 35–40 °C) and aggregate into bundles to form a network. This physical crosslinking is reversible, with melting occurring above 85 °C. This thermal behavior facilitates “smart” applications in bioprinting and injectable therapies where temperature controls the material’s state [123,124].

The hydrogels formed by agarose exhibit exceptional and tunable stiffness, with the Young’s modulus directly correlated with concentration, ranging from approximately 10 kPa to over 500 kPa. High-concentration gels (up to 4–5%) can approach the stiffness of the native pericellular matrix (PCM) of articular cartilage. This provides a mechanical environment that maintains the chondrocyte phenotype more effectively than softer hydrogels like fibrin. Biologically, agarose differs from protein-based polymers; it is bio-inert and lacks native cell adhesion motifs such as RGD. While this “blank-slate” nature prevents immunogenic rejection, it also prevents cell spreading unless the material is chemically modified or blended with bioactive polymers. In cartilage engineering, this lack of adhesion is beneficial as it supports the spherical cell morphology conducive to chondrogenesis [125,126,127].

A key feature of agarose is its resistance to enzymatic degradation in mammals, primarily because mammalian cells lack agarase. Consequently, the material degrades very slowly via hydrolytic mechanisms or dissolution, providing critical long-term structural support while chondrocytes synthesize functional ECM—a process that requires months. In terms of applications, agarose scaffolds successfully preserve the chondrocyte phenotype and support the accumulation of hyaline cartilage markers, such as sulfated glycosaminoglycans (sGAGs) and Collagen II. For bone regeneration, composite scaffolds of agarose and hydroxyapatite or bioactive glass show promise, as agarose provides a matrix for HA dispersion to create constructs with enhanced rigidity and osteoconductivity. Additionally, agarose is frequently blended with alginate or collagen to improve bioink printability, using its rapid gelation to hold the shape of printed constructs while secondary crosslinking occurs [126,127,128].

**Figure 1:**
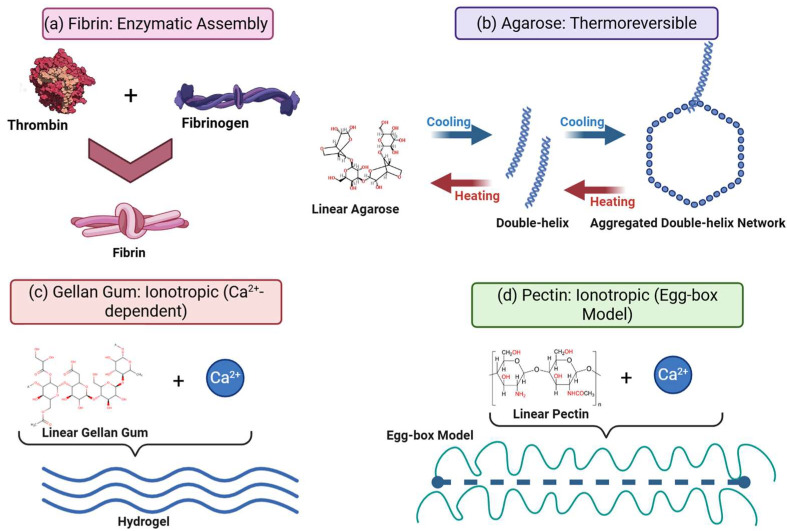
Different gelation techniques for natural polymers. Created in BioRender. Calin et al. (2026) https://BioRender.com/zq8ecvz (accessed on 28 March 2026).

Pectin is a complex heteropolysaccharide derived from the cell walls of terrestrial plants like citrus peels and apple pomace and is emerging as a potent biomaterial due to its specific gelation mechanism and intrinsic bioactivity. Structurally, pectin contains a backbone of galacturonic acid residues. Low-methoxyl (LM) pectin gels in the presence of calcium ions (Ca^2+^) via the “egg-box” model (Figure 1), a mechanism similar to alginate but with distinct biological effects due to its specific side chains or “hairy” regions. While pectin hydrogels are generally soft, they can be significantly reinforced; composites such as pectin-grafted polycaprolactone (Pectin-g-PCL) can achieve compressive moduli ranging from roughly 3 kPa up to 5 MPa, effectively bridging the gap between soft hydrogels and harder tissue scaffolds [129,130].

Unlike the bio-inert agarose, pectin exhibits intrinsic anti-inflammatory and immunoregulatory properties. It has been shown to downregulate the secretion of pro-inflammatory cytokines, such as TNF-α, in macrophages, thereby promoting a pro-healing microenvironment suitable for tissue regeneration. Additionally, pectin is widely used in wound dressings due to its high water-holding capacity, which allows it to absorb wound exudates while maintaining a moist environment that accelerates re-epithelialization and granulation tissue formation [129].

A detailed comparison of these polymers is provided in Table 1, integrating quantitative parameters from multiple studies.

**Table 1:** Comparison of natural polymers, their physical properties and morphology.

Polymer	Source	Tensile/ Compressive Strength	Degradation Rate	Porosity (%)	Cell Viability (%)	Benefits	Limitations	Reference
Collagen	Mammalian connective tissue	50–500 MPa (tensile)	4–6 weeks (non-crosslinked)	70–95	>90	High bioactivity, supports osteogenesis	Immunogenicity risk, fast degradation	[131,132,133]
Gelatin	Denatured collagen	10–100 kPa (hydrogel modulus)	2–3 weeks	80–98	>85	Easy processing, bioactive	Low mechanical strength	[134,135]
Chitosan	Crustacean shells	2–12 MPa (blends)	Weeks–months	65–85	>90	Antibacterial, osteoconductive	Poor solubility at neutral pH	[136,137,138,139]
Alginate	Brown algae	<1 MPa	Weeks–months	80–95	70–90	Mild gelation, printable	Lacks adhesion motifs	[140,141,142]
Silk fibroin	Silkworm cocoons	Up to 740 MPa	Months–years	60–85	>90	High strength, tunable degradation	Hydrophobicity	[143,144]
Hyaluronic acid	ECM component	Low modulus	<24 h (native)	85–98	>95	ECM mimicry, promotes angiogenesis	Rapid degradation	[145,146,147]
Fibrin	Blood plasma (fibrinogen + thrombin)	Low; strain-stiffening	Rapid (days–weeks); proteolytic (plasmin/MMP); highly cell-responsive	High (>90%)	>95%	Native RGD motifs; intrinsic bioactivity; chemotactic; remodelable	Mechanically weak (<30 kPa); degrades too fast for long-term support	[120,148]
Agarose	Red algae (*Rhodophyceae* such as *Gelidium*)	~25 kPa (2%)	Very slow/non-degradable in mammals (lack agarases); stable for months	Nanoporous (decreases with conc., e.g., <100 nm at 3%)	Good (50–75%)	High stiffness (cartilage mimic); shape retention; thermoreversible	Inert (no adhesion); non-degradable (foreign body risk); nanoporosity limits integration	[149]
Pectin	Plant cell walls (citrus/apple peel)	6–100 kPa (Ca-crosslinked)	Moderate/tunable (weeks); enzymatic (pectinase/lysozyme); Mass loss ~30% in 3 days (blends)	~50%	>90%	Versatile gelation (pH/ions); low cost; high tensile strength in blends	Ionic instability (Ca^2+^ loss); brittle if over-crosslinked; variable source properties	[150]
Carrageenan	Red algae (*Rhodophyceae*)	Compressive modulus: 30–60 kPa (hybrid); tensile: ~1.3 MPa (double network)	Tunable (weeks); Enzymatic (carrageenase) or hydrolytic	70–90%	>90%	Thermoreversible gelation, mimics sulfated GAGs, antiviral/anti-inflammatory properties	Brittleness in pure form; potential inflammation from low-MW byproducts	[151,152,153]
Decellularized ECM (dECM)	Porcine/bovine tissues (heart, skin, bone)	Flexural: 90–130 MPa (bone composites)	Weeks to months	80–95%	>95%	Perfect biochemical mimicry, retains cryptic peptides/growth factors, tissue-specific cues	Batch-to-batch variability; risk of residual immunogens (α-Gal); complex processing	[154,155,156]
Keratin	Wool, human hair	Compressive: 0.2–1.2 MPa (reinforced with HA)	Proteolytic degradation (weeks); tunable via crosslinking	75–85%	>90%	Intrinsic cell binding motifs (LDV, EDS); osteoinductive; high cysteine content	Fragile in pure form; extensive extraction processing required	[157,158]
Ulvan	Green algae (*Ulva* species)	Modulus: ~4 MPa (PCL blends); weak pure hydrogels	Moderate (weeks); depends on degree of crosslinking	80–90%	>85%	Structurally similar to heparin; promotes osteogenic differentiation; renewable resource	Seasonal chemical variability; difficult extraction/purification; low gel strength	[159,160]
Xanthan gum	*Xanthomonas campestris* fermentation	Compressive modulus: 3–200 kPa (ionic crosslinking)	Slow in vivo degradation; highly stable	>90%	>95%	Excellent rheological modifier (shear-thinning); bio-inert backbone; tunable stiffness	Lack of intrinsic cell adhesion sites; susceptible to microbial contamination if not sterile	[161,162,163]

### 2.3. 3D and 4D Printing, Implant Coatings, and Advanced Fabrication Approaches

**Figure 2:**
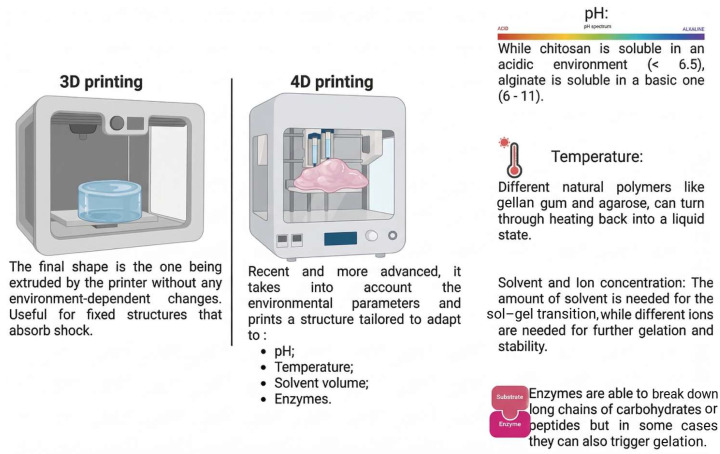
3D versus 4D printing and the uniqueness of smart hydrogels. Created in BioRender. Calin et al. (2026) https://BioRender.com/p3ve6ap (accesed on 28 March 2026).

Extrusion bioprinting is the most popular approach for natural polymers such as alginate and GelMA. It can withstand high-viscosity inks but causes shear stress on cells. Shear-thinning qualities are critical for printability; for example, alginate/gelatin mixes are commonly employed to balance viscosity and cell viability (Figure 2). The “biofabrication window” notion is important here: balancing print fidelity (high viscosity/crosslinking) with cell survival (low shear/soft matrix) [164,165]. Inkjet and stereolithography (SLA/DLP) provide better resolution. Light-sensitive natural polymers (e.g., GelMA and HAMA) are polymerized using photoinitiators such as LAP or ruthenium under visible or UV light. SLA allows for the creation of complex vascular networks inside hydrogels, which is a significant limitation of extrusion approaches [166].

The emergence of additive manufacturing has revolutionized the production of polymeric scaffolds, facilitating meticulous regulation of pore geometry, spatial arrangement of bioactive agents, and mechanical gradients (Table 2). Rheological tuning and crosslinking techniques have made it possible to print with naturally occurring polymers like alginate, gelatin, chitosan, and silk fibroin. For instance, gelatin methacryloyl (GelMA) bioinks have shear-thinning properties that make them perfect for extrusion printing. After printing, UV crosslinking gives them compressive moduli of 20–60 kPa, which is good for soft tissue applications [167,168,169,170]. Adding nano-hydroxyapatite to chitosan/alginate mixes for bone tissue engineering can make them stronger while keeping their porosities high. Experimental data indicate that the compressive strength of these scaffolds can be precisely engineered by adjusting the HAp content [171]. Increasing HAp from 17% to 35% wt has been shown to raise the compressive strength from 1 MPa to 3.2 MPa, which falls within the physiological range of human cancellous (spongy) bone (2–12 MPa) [172]. Furthermore, the Young’s modulus of such composite scaffolds, measured at approximately 132 MPa (0.13 GPa), is well-matched to the modulus of natural cancellous bone (0.05–0.5 GPa).

This mechanical matching ensures that the scaffold can maintain its structure during the critical early stages of bone healing, preventing premature displacement under physiological loads. Beyond mechanical support, the presence of HAp increases the local concentration of Ca^2+^ and PO_4_^3−^ ions, which stimulates osteoblast proliferation and the maturation of the mineralized matrix. When combined with high interconnected porosity (often > 70%), these scaffolds allow for deep cellular infiltration and the necessary transport of nutrients and metabolic waste required for functional bone regeneration [13,173,174].

The role of AM in controlling scaffold microarchitecture is intrinsically linked to the mechanical environment of the regenerating tissue. In bone tissue engineering, for instance, the meso- and microscale design dictates the bulk mechanical properties, such as the Young’s modulus and compressive strength [175]. By adjusting parameters like the crosshatch pattern, layer height, and fiber diameter, AM allows for the production of scaffolds that match the stiffness of the surrounding cortical or cancellous bone [51]. This hierarchical control is essential because the mechanical stress experienced by cells at the scaffold interface dictates differentiation and tissue maturation, a concept known as mechanoadaptation [13,176].

Specific AM techniques offer distinct advantages depending on the material and application. Stereolithography (SLA) uses photosensitive resins to create high-resolution hydrogel scaffolds for central nervous system (CNS) repair, while Fused Deposition Modeling (FDM) is frequently utilized for thermoplastic polymers like PLA or PCL in load-bearing applications [177]. Techniques such as Dynamic Baseline Restorative Printing (DBRP) and Freeze-FRESH bioprinting further extend these capabilities, enabling the fabrication of scaffolds with hierarchical porosity that mimics the complex vascular networks necessary for oxygen and nutrient transport in thick tissue constructs [178]. These advances facilitate a “bottom-up” paradigm where cellularized building blocks assemble into larger tissue-like structures that recapitulate native hierarchy [179].

**Table 2:** Recent techniques used for molding and shaping natural polymers.

Technique	Typical Materials	Advantages	Restraints	References
Electrospinning	Collagen, chitosan, silk blends	ECM-like nanofibers; large surface area	Limited cell infiltration unless porous	[180,181,182]
3D bioprinting	GelMA, alginate, GelMA–alginate hybrids	Spatial control of cells and growth factors	Bioink rheology and crosslinking constraints	[183,184]
Injectable in situ gels	Alginate, thermogelling chitosan/gelatin	Minimal invasiveness; defect conformation	Controlling gelation and retention in vivo	[185,186]
Freeze-drying/porogen leaching	Collagen, gelatin	High porosity; simple	Poor mechanical strength; batch variability	[187,188,189]
4D printing/stimuli-responsive	Smart hydrogels, modified natural polymers	Dynamic remodeling; shape change after implantation	Early technology; limited biological validation	[190,191,192]
Melt electrowriting (MEW)	PCL/chitosan blends, PCL/gelatin	Solvent-free fabrication; highly ordered microfibrous architecture (2–50 µm); combines porosity with mechanical resilience	Limited to thermally stable polymers; requires specialized equipment; slower vertical build rates than extrusion	[193,194]
Digital Light Processing (DLP)	GelMA, methacrylated silk (SilMA), PEGDA/alginate	Ultra-high resolution (~25 µm); rapid layer-by-layer curing; prints complex geometries without support structures	Requires photo-curable moieties (methacrylation); potential cytotoxicity of photo-initiators; limited material viscosity range	[195,196,197]
Microfluidics	Alginate, gelatin, silk fibroin	Monodisperse microgel production; high control over internal architecture (core–shell); protects encapsulated cells from shear	Low throughput for macroscale scaffold production; complex device fabrication; potential channel clogging	[198,199,200]
Gas foaming	Alginate, gelatin, chitosan	Solvent-free and low-temperature process; creates highly interconnected pores; preserves bioactive agents	Lack of precise control over pore architecture; formation of non-porous skin layer; requires high-pressure equipment	[201,202,203]
Coaxial bioprinting	Alginate/pluronic, GelMA/alginate	Fabrication of vessel-like (tubular) structures; enhanced cell viability via core–shell shielding; multi-material deposition	Complex rheological matching required between core and shell; lower resolution than DLP; limited to continuous filaments	[204,205,206]

In cartilage engineering, HA-based hydrogels infused with magnetically responsive nanoparticles have been 4D-printed to fabricate constructs capable of remote stimulation to modify stiffness, thereby facilitating chondrogenic differentiation in vitro [207,208].

**Figure 3:**
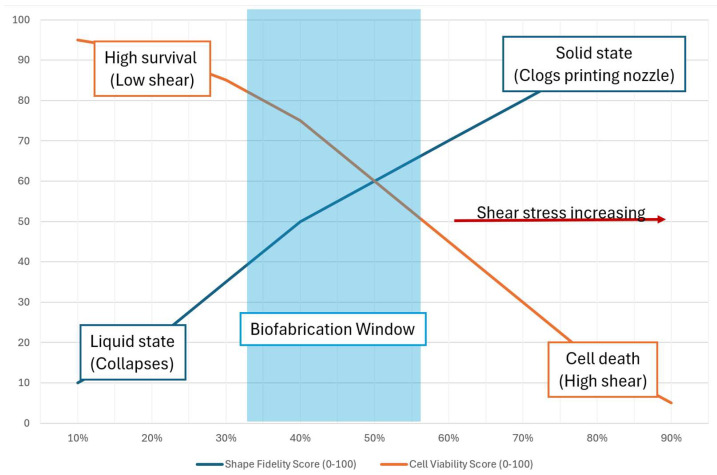
The ideal parameters to be taken into account in bioprinting.

Natural polymers often swell and shrink when exposed to water or solvents. By printing materials with varying swelling ratios (e.g., high-swelling alginate vs. low-swelling cellulose or silk) in a bilayer structure, differential expansion causes bending, folding, or twisting when hydrated (Figure 3). In vivo, a self-folding tracheal stent consisting of a hydrogel bilayer was demonstrated to coil into a tubular form when in contact with physiological fluids in a rabbit model, allowing for minimally invasive administration [209].

Gelatin exhibits sol–gel transitions when the temperature varies. More advanced techniques combine Poly(N-isopropylacrylamide) (PNIPAAm) segments into natural polymer backbones. Below the lower critical solution temperature (LCST), the polymer is hydrophilic and swollen; above it (for example, at 37 °C), it becomes hydrophobic and collapses, causing shape change. Silk fibroin–PNIPAAm composites had >90% shape recovery ratios, suggesting that large scaffolds can be compressed for injection and subsequently extended in situ to fill bone defects [210,211].

Polymers such as chitosan (cationic) and alginate (anionic) react to pH changes by ionizing or deionizing, affecting charge repulsion and hydration. In the acidic tumor microenvironment (pH ~6.5), pH-sensitive hydrogels can release medicines or expose bioactive ligands. 4D-printed chitosan-based devices have been engineered to contract or expand in the gastrointestinal tract (at different pH levels) to function as mucoadhesive patches [212]. The incorporation of iron oxide nanoparticles into alginate or collagen bioinks enables remote actuation. An external magnetic field aligns the magnetic domains or produces heat (hyperthermia), which causes shape change or medication release. Magnetically sensitive 4D-printed constructs have been utilized to mechanically excite cells in vitro, promoting chondrogenic differentiation of stem cells by simulating joint dynamic stress (Table 3) [213,214].

**Table 3:** 4D printing mechanisms for natural polymers and tunable parameters.

Stimulus	Responsive Material	Mechanism of Action	Biomedical Application	Reference
**Water/Ion**	Alginate/Cellulose Bilayers	Differential swelling/anisotropic expansion	Self-folding tracheal stents, vascular grafts	[215,216]
**Temperature**	Gelatin–PNIPAAm, Silk–PNIPAAm	Coil-to-globule transition around LCST (~32–37 °C)	Injectable bone scaffolds, smart wound dressings	[217,218,219]
**pH**	Chitosan, Alginate	Protonation/deprotonation causing electrostatic repulsion/attraction	Smart wound dressings sensitive to infection	[220,221]
**Magnetic**	Alginate + Fe_3_O_4_ NPs	Magneto-mechanical alignment or induction heating	Remote actuation, mechanotransduction stimulation	[222]
**Enzymatic**	Collagen, HA (MMP-Sensitive)	Cleavage of crosslinks by specific enzymes (e.g., MMPs)	Cell-mediated remodeling, degrading scaffolds	[223,224]
**Light (UV/IR)**	Methacrylated Silk, GelMA–Gold Nanorods	Photo-cleavage of crosslinks or photothermal heating inducing phase transition/shrinkage	On-demand drug release, remote actuation of soft grippers, spatiotemporal control of cell adhesion	[225,226]
**Electric Field**	Chitosan–Graphene, Alginate–CNT	Electro-osmotic ion migration causing asymmetric swelling/bending	Artificial muscles, electro-stimulated nerve regeneration, smart drug-release devices	[227,228]
**Multi-Stimuli (pH + Temp)**	Chitosan–PNIPAAm, Alginate–Pluronic	Dual-response logic (e.g., sol–gel at body temp. + swelling at acidic pH)	Tumor-targeted delivery (acidic/warm microenvironment), complex shape-morphing scaffolds	[229,230,231]
**Glucose**	Concanavalin A-Modified Alginate/Chitosan	Competitive binding of glucose disrupts polymer crosslinks, increasing porosity	Self-regulated insulin delivery systems (artificial pancreas), diabetes management	[232,233]
**Reactive Oxygen Species (ROS)**	Thioketal-Modified Keratin or Hyaluronic Acid	Cleavage of ROS-sensitive linkers triggers degradation or drug release	Targeting inflamed tissues (e.g., chronic wounds, cardiac infarction) with antioxidant release	[234,235]

Scaffolds containing specific peptide sequences (e.g., MMP-sensitive crosslinks) can degrade or change shape in the presence of enzymes secreted by remodeling cells. This allows the scaffold to “sense” the rate of tissue regeneration and adapt its support accordingly.

One of the best ways to make metallic and ceramic implants more biointegrated is to change their surfaces with polymeric coatings. Chitosan coatings on titanium alloys have demonstrated a reduction in bacterial adhesion by up to 70% relative to uncoated controls, while concurrently increasing osteoblast proliferation by 40% [236,237,238]. In a similar way, collagen-coated hydroxyapatite implants put into rabbit femoral defects showed much higher bone-to-implant contact ratios (68% vs. 42% in uncoated controls) after 12 weeks [239]. Silk fibroin coatings sprayed with plasma have also been shown to help fibroblasts stick to them and change the way macrophages work to make them more pro-healing M2 types, which lowers chronic inflammation [240,241].

Electrospinning natural polymers is another way to make ECM-mimicking nanofibers with a high surface area-to-volume ratio, which is important for guiding cell attachment and movement. Chitosan/polycaprolactone (PCL) nanofibers, measuring 200–500 nm in diameter, have been demonstrated to enhance neurite outgrowth by 45% in dorsal root ganglion cultures, indicating their potential in neural regeneration [23,242,243]. Electrospun gelatin mats crosslinked with genipin have tensile strengths of 1–3 MPa, which makes them good for skin substitutes. They also let water vapor through at a rate that keeps wounds from drying out [244,245].

## 3. The Use of Injectable Gels and Scaffolds in Experimental Therapy

Injectable polymeric systems have become useful for delivering cells, growth factors, or bioactive molecules directly to defects that are not perfectly shaped, so there is no need for preformed implants. Polymers that come from nature are especially good for these kinds of systems because they are intrinsically bioactive and can gel in mild physiological conditions (Table 4).

Chitosan-based injectable hydrogels, frequently crosslinked with β-glycerophosphate, experience sol–gel transition at physiological temperature, resulting in viscoelastic networks with storage moduli attaining up to 1000 Pa within minutes. These gels have been evaluated in vivo for the delivery of mesenchymal stem cells into osteochondral defects, achieving up to 85% defect filling with hyaline-like cartilage after 12 weeks in rabbit models [246,247,248]. Alginate-based injectable systems have shown promise in myocardial repair, as slow-release vascular endothelial growth factor (VEGF)-loaded gels enhanced left ventricular ejection fraction by about 9% compared to saline controls in porcine infarct models [249]. HA hydrogels are used in clinical settings for minimally invasive cartilage repair because they are naturally viscoelastic. Crosslinked HA–tyramine systems catalyzed by horseradish peroxidase gel in less than 60 s and have compressive moduli between 15 and 30 kPa. In pilot clinical studies, intra-articular injection of HA-based scaffolds resulted in 25–30% enhancements in International Knee Documentation Committee (IKDC) scores at the six-month follow-up [250].

Self-healing hydrogels represent an innovative category of injectable systems that utilize reversible covalent bonds, specifically Schiff base linkages, between aldehyde-functionalized polysaccharides and amino-bearing proteins. These designs are strong enough to handle repeated mechanical stress. For instance, alginate–gelatin hydrogels with self-healing properties could regain more than 90% of their original storage modulus within five minutes of being mechanically damaged. This meant that they could be deformed over and over again without losing their structural integrity [251]. In situ-forming scaffolds, like silk fibroin sol–gel systems or fibrin–collagen composites, are even more flexible because they can be injected as liquids and then harden in place. Thermoresponsive silk fibroin blends solidify in less than 10 min at 37 °C and can reach compressive strengths of up to 2 MPa, making them very promising for filling bone defects with little risk of infection [252]. Fibrin-based in situ-forming scaffolds have also been utilized in craniofacial repair, demonstrating 70% enhanced vascularization compared to collagen-only controls in murine models [253].

High porosity (>90%) and connectedness are required for nutrition transport and vascular infiltration. Increased porosity usually lowers mechanical strength. Granular hydrogels (jammed microgels) provide a unique approach by decoupling porosity and stiffness. Packing stiff microgels together results in a construct with high local stiffness (promoting osteogenesis) and high macroporosity (facilitating migration), which is difficult to achieve in bulk hydrogels [254]. In collagen scaffolds, pore size affects migratory speed. Larger holes (>150 µm) can diminish cell–scaffold contact guidance, whereas smaller pores may physically confine cells unless they can destroy the matrix [255].

The degradation rate must also synchronize with neo-tissue development. Rapid breakdown of non-crosslinked collagen (4–6 weeks) can lead to premature mechanical failure, whereas too stable silk fibroin (months to years) can inhibit bone rebuilding by acting as a stress barrier. Natural polymers break down into non-toxic amino acids and carbohydrates that can be digested. However, the accumulation of acidic byproducts from composite materials (e.g., PLGA blends) can cause local inflammation, which must be handled with buffering agents such as hydroxyapatite [256].

### 3.1. Bone Regeneration

Bone regeneration is one of the most studied uses of injectable biomaterials. Mechanical performance is crucial for defect stability, and recent advancements underscore substantial enhancements via composite formulations. A double-network hydrogel made of poly(vinyl alcohol) and bioactive glass microspheres had a compressive strength of about 34 MPa, a modulus of about 0.8 MPa, and a fracture energy of about 40 kJ/m^2^, which are values that are close to what trabecular bone needs [257]. GelMA hydrogels fortified with hydroxyapatite microspheres demonstrated peak compressive stresses of approximately 138 ± 5 kPa at a 1% filler content. Concurrently, an increase in GelMA concentration from 5% to 15% elevated the compressive modulus from around 4.2 to 50 kPa [258,259,260]. In vivo, alginate–hydroxyapatite hydrogels resulted in 65% new bone formation in calvarial defects, compared to 40% for pristine alginate [236]. Fibrin–hydroxyapatite composites enhanced osteoid deposition by nearly 1.8-fold in rat models [239]. In bone tissue engineering, pectin promotes the nucleation of mineral phases (hydroxyapatite) when exposed to physiological fluids, enhancing osteointegration. Blends with chitosan or bioactive glass further enhance this osteoconductive potential, creating scaffolds that mimic the mineralized matrix of bone [129].

### 3.2. Cartilage Repair

For cartilage applications, injectable gels must endure repetitive mechanical stresses while preserving a hydrated environment that facilitates chondrogenesis. Quantitative studies indicate that the compressive moduli of nano-composite hydrogels intended for osteochondral repair vary from 0.4 MPa to over 70 MPa, contingent upon filler composition and polymer crosslinking methodology [261,262]. For example, polycaprolactone–hydroxyapatite (PCL–HA) composites had compressive moduli greater than 70 MPa, while gelatin methacrylate–IL-4 composites were less stiff but better for chondrogenic differentiation. In vivo, cellulose–polyacrylamide composite hydrogels implanted in rabbit femoral defects withstood compressive stresses of 3–10 MPa under cyclic compression after 90–120 days, values that closely resemble the performance of native cartilage [263,264]. Dual-network hydrogels modified with strontium ions enhanced chondrogenesis of bone marrow-derived stem cells and preserved structural integrity under cyclic shear, highlighting the promise of multifunctional bioactive systems [265].

### 3.3. Nerve Regeneration

Injectable hydrogels are progressively being modified for peripheral nerve regeneration, necessitating the provision of aligned topographical cues and biochemical support for axonal outgrowth. In vitro studies show that aligned electrospun gelatin–chitosan scaffolds enhance neurite length by 1.5 to 2 times compared to isotropic controls [266]. In vivo, hyaluronic acid granular hydrogel conduits bridging 10 mm sciatic nerve gaps in rodents restored compound muscle action potential amplitudes to levels comparable with autologous nerve grafts, while significantly increasing conduction velocity within 12 weeks [267]. Moreover, silk fibroin–hyaluronic acid hybrids have demonstrated the ability to promote Schwann cell proliferation and remyelination, thereby expediting the functional recovery of motor coordination in rat sciatic nerve models [268,269]. Fibrin conduits offer a soft environment that promotes nerve regeneration. When aligned by electrospinning or magnetic orientation, fibrin fibers can provide contact guidance for bridging nerve gaps, providing a bioresorbable alternative to autografts [270].

### 3.4. Cardiac Applications

Cardiac tissue constitutes a highly dynamic milieu in which injectable scaffolds must withstand pulsatile forces while facilitating neovascularization and contractile recovery. Meta-analyses of animal models of myocardial infarction indicate that combined hydrogel–cell or hydrogel–growth factor therapies enhance left ventricular ejection fraction by 8.9% in rats and up to 16.5% in mice, with fractional shortening improvements of approximately 6.3% and 5.7%, respectively [271,272,273,274]. In porcine models, injection of a theranostic hydrogel enhanced regional strain, resulting in a 52.7% increase in radial strain and a 44.1% increase in circumferential strain in the infarcted myocardium compared to baseline measurements, while untreated controls exhibited a progressive decline [275,276,277,278]. Alginate hydrogels diminished infarct size by approximately 53% and augmented capillary density by more than 150% in border zones, concurrently reducing apoptotic cell fractions by about 40% in rodent models [279,280,281]. These data collectively illustrate that injectable polymeric matrices not only maintain wall thickness but also provide significant functional advantages in cardiac repair.

Therapies for myocardial infarction (MI) aim to mitigate adverse left ventricular (LV) remodeling (characterized by wall thinning and dilation) which compromises cardiac output [282]. Injectable hydrogels provide structural reinforcement and a pro-regenerative environment.

PNIPAAm-based hydrogels are liquid at room temperature and undergo a sol-to-gel transition at a lower critical solution temperature (LCST) of approximately 32 °C [283]. In rat models, intramyocardial injection of PNIPAAm (Gel A and Gel B) immediately post-MI inhibited LV expansion and improved contractility. Notably, the slower-degrading Gel B provided superior functional repair [284]. In large-animal models (sheep), NIPAAm-mPEGMA copolymers act as ROS scavengers [285]. PEG side chains are cleaved by ROS, reducing hydroxyl radical and superoxide levels by 88% in the border zone. This quenching inhibits matrix metalloproteinase-2 (MMP-2) activity, preventing the degradation of essential contractile proteins like troponin I and titin [21].

Alginate hydrogels serve as passive ventricular assist devices by increasing wall thickness and decreasing peak wall stress. In swine models of heart failure, Algisyl-LVR increased the ejection fraction from 30.5% to 42.4% over 16 weeks [286]. Clinical results from the AUGMENT-HF trial showed that patients receiving these injections alongside standard medical therapy (SMT) had sustained 1-year improvements in peak VO_2_ and 6 min walk test (6MWT) distance compared to SMT alone [21].

Hydrogels act as moisture donors that hydrate dry necrotic tissue, enabling autolytic debridement—a selective process where endogenous enzymes (collagenases, elastases, and MMPs) liquefy dead tissue [287,288]. This environment also facilitates macrophage-led phagocytic cleanup. In chronic wounds, thiolated chitosan (TCS) hydrogels can chelate Zn^2+^ ions to inhibit excessive MMP and myeloperoxidase (MPO) activity (up to 98%), protecting new tissue from premature degradation [289].

Nitric oxide (NO)-releasing hydrogels provide a multi-target antimicrobial strategy that disrupts bacterial DNA, proteins, and lipid membranes. The bactericidal potency is amplified by the in situ reaction between NO and superoxide (O_2_^−^) to form peroxynitrite (ONOO^−^) [290,291].

Peroxynitrite is a highly reactive oxidant that crosses bacterial membranes to initiate lipid peroxidation and destroy cellular structures. In gelatin-based (GH/GelSNO) systems, complete eradication of *E. coli* and *S. aureus* was achieved at NO concentrations of 0.39 µmol/mL and 0.58 µmol/mL, respectively. These systems also induce biofilm dispersal, making bacteria more susceptible to immune clearance [252,290,292].

### 3.5. Soft Tissue and Wound Healing

To heal wounds and regenerate soft tissue, scaffolds need to be both elastic and bioactive. Injectable collagen–elastin composites demonstrate a strain-to-failure exceeding 80%, closely resembling the mechanical properties of dermal tissue. In preclinical wound models, these scaffolds expedited re-epithelialization by as much as 35% relative to collagen-only controls [251,293]. Natural gum polysaccharides, including guar and xanthan, have been engineered into thermosensitive injectable hydrogels that facilitate the sustained release of antibiotics or growth factors over a duration of 10–14 days, while concurrently promoting granulation tissue formation and angiogenesis [294,295,296]. In clinical settings, HA-based injectable hydrogels have demonstrated a 25–40% decrease in healing time for chronic ulcers when compared to traditional dressings. By interacting with cell-surface receptors such as CD44 and RHAMM, HA stimulates the migration and proliferation of fibroblasts and keratinocytes, which are essential for re-epithelialization. Furthermore, HA promotes angiogenesis by upregulating vascular endothelial growth factor (VEGF) expression. These combined mechanisms contribute to a reported reduction in chronic ulcer healing time by up to 40% compared to standard saline-based dressings, particularly in recalcitrant cases like diabetic foot ulcers and venous leg ulcers, where moisture retention and controlled inflammation are critical for closure [251].

Fibrin promotes angiogenesis. It has native binding sites for growth factors (e.g., VEGF and bFGF) and integrins, which enhance endothelial cell migration and capillary tube formation. This makes fibrin a necessary component in vascularized tissue structures [297]. Fibrin–agarose composites are effective alternatives for the cornea and skin, combining fibrin’s bioactivity with agarose’s mechanical resilience to generate durable and cell-friendly structures [298].

**Table 4:** Versatile uses for injectable natural polymers, crosslinks and composites.

Application	System/Composition	Gelation/ Crosslinking	Mechanical Properties	Biological/Clinical Outcomes	References
Bone regeneration	GelMA + HA microspheres	Photocrosslinking	Compressive stress: ~138 ± 5 kPa; modulus: ↑ from 4.2 to 50 kPa (5–15% GelMA)	Enhanced MSC osteogenesis	[299]
	Alginate–HA hydrogel	Ionic (Ca^2+^)	50–70 kPa	65% new bone vs. 40% pristine alginate (rat calvarial defect)	[236]
	PVA + bioactive glass	Dual-network	Compressive strength: ~34 MPa; modulus: ~0.8 MPa	Improved bone-like mechanical fidelity	[257]
Osteochondral regeneration	Bilayered chitosan/xanthan gum + biphasic calcium phosphate	Freeze-drying/ionic crosslinking	Compressive strength; ~1–5 MPa (gradient stiffness)	Integrated repair of bone-cartilage interface; support of subchondral bone formation	[300]
Cartilage repair	HA–tyramine	Enzymatic (HRP/H_2_O_2_)	Compressive modulus: 15–30 kPa	IKDC ↑ 25–30% at 6 months (clinical)	[301,302]
	Nano-composite hydrogels (PCL–HA, GelMA–IL-4, etc.)	Photocrosslinked/ionic	0.4–70 MPa depending on filler	Promoted BMSC chondrogenesis, improved load-bearing	[303,304,305]
	Cellulose–PAM composite	Dual-network	3–10 MPa under cyclic load (90–120 days)	Maintained function in rabbit osteochondral repair	[264,306,307]
Intervertebral disc repair	Chitosan–hyaluronic acid/silk–PU blends	Thermosensitive (sol–gel at 37 °C)/chemical	Compressive modulus: 10–50 kPa (matches nucleus pulposus); fatigue-resistant	Restoration of Disc Height Index (DHI) and MRI signal intensity in rabbit models; prevents further degeneration	[308]
Nerve regeneration	Electrospun gelatin–chitosan	Injectable aligned scaffold	Not reported	1.5–2× longer neurites in vitro; motor recovery in 8 weeks (rat sciatic)	[266]
	HA granular hydrogel	Injectable granular packing	Not reported	Restored CMAP amplitude; ↑ conduction velocity after 12 weeks	[267]
	Silk fibroin–HA hybrid	Self-assembling	Not reported	Enhanced Schwann cell proliferation; improved remyelination	[267]
Advanced nerve repair	Conductive chitosan/polypyrrole or aligned fibrin	In situ polymerization/magnetic alignment	Conductivity: ~10^−3^ S/cm; modulus: ~1 kPa (soft tissue match)	Restored nerve conduction velocity and CMAP to autograft levels at 12 weeks; enhanced myelination	[309]
Cardiac repair	Alginate (VEGF-loaded)	Ionic	Not reported	LVEF ↑ 9% in porcine MI model	[249]
	Chitosan–gelatin + IGF-1	Thermosensitive	Not reported	↓ infarct size by 22%; ↑ angiogenesis in rats	[271]
	Hydrogel–cell/growth factor composites	Various	Not reported	Meta-analysis: LVEF ↑ 8.9% (rats), ↑ 16.5% (mice); FS ↑ ~6%	[272,274,310]
	Theranostic hydrogel	Injectable/responsive	Not reported	Radial strain ↑ 52.7%; circumferential strain ↑ 44.1% (porcine MI)	[311,312,313,314]
Soft tissue/wound healing	Collagen–elastin blend	Self-assembly	Strain-to-failure: >80%	35% faster re-epithelialization vs. collagen	[251]
	HA injectable hydrogel	Crosslinked	Not reported	25–40% faster ulcer healing vs. controls	[251]
	Natural gum-based gels	Thermosensitive/ionic	Not reported	Sustained release up to 14 days; improved angiogenesis	[294]
Hemostasis	Carboxymethyl chitosan + oxidized dextran/gelatin	Schiff base reaction (rapid self-healing)	Storage modulus: >1 kPa; adhesive strength: >10 kPa	Stopped bleeding in rat liver trauma in <40 s (vs. 167 s for gauze); reduced total blood loss by >80%	[315]

In practice, the mechanical properties of GG hydrogels can be tuned to match the nucleus pulposus of the intervertebral disc, making them capable of restoring disc height and biomechanics in loaded disc models. GG exhibits mucoadhesive properties and stability in the acidic environment of the stomach, making it an excellent vehicle for controlled oral and nasal drug delivery systems. In the realm of 3D bioprinting, GG solutions exhibit shear-thinning behavior and rapid ionic crosslinking, making them excellent candidates for bioinks. They often serve as sacrificial support structures or as the primary structural matrix in complex printed tissues [316,317].

For injectable constructs that undergo significant deformation during needle extrusion, hydrogels containing reversible Schiff base linkages, phenylboronate esters, or Diels–Alder adducts exhibit self-healing behavior that permits mechanical strength recovery following shear-induced network disruption. Also, by facilitating viscoelastic tuning, these dynamic interactions allow materials to replicate the stress-relaxation profiles of native tissues like myocardium or cartilage. The functional capabilities of natural polymers are further expanded by stimuli-responsive networks: HA-based gels with redox-cleavable crosslinkers degrade selectively in highly inflammatory environments, chitosan-based hydrogels modified with thermo-responsive segments change from liquid to solid in vivo, and alginate matrices integrated with magnetic nanoparticles can experience remote stiffness modulation to improve mechanotransduction during chondrogenesis. These systems show that natural polymers can be designed to function as more than just passive scaffolds.

## 4. Translational and Regulatory Considerations

Even though there is strong preclinical evidence for injectable gels and in situ-forming systems, there are still a number of problems that make it hard to use them widely in clinical practice. Polymers that come from nature can vary a lot from batch to batch, which makes it hard to reproduce them and get them approved by the government.

Sterilization processes can also change the physical or biochemical properties of these materials. High-energy gamma radiation (usually 25 kGy) frequently induces polypeptide chain scission in collagen and chitosan, resulting in considerable reductions in molecular weight and mechanical strength. Gamma irradiation can lower viscosity in chitosan by more than 90% and damage the polymer backbone, influencing medication-release characteristics [318]. However, it is effective and leaves no trace. Ethylene oxide (EtO) is typically more favorable to mechanical qualities, although it can leave hazardous residues (ethylene chlorohydrin). It works by alkylating amino acid side chains, which might affect cell attachment sites (bioactivity). Comparative investigations on collagen scaffolds have demonstrated that gamma irradiation disrupts the triple-helix structure, resulting in fast deterioration, whereas EtO retains structure but necessitates prolonged aeration to eliminate harmful residues. EtO can alkylate amino, carboxyl, and hydroxyl groups within the polymer backbone; such modifications may sterically hinder cell-surface receptor interactions (e.g., integrin binding to RGD motifs), thereby diminishing the scaffold’s bioactivity. Consequently, recent trends have shifted toward supercritical CO_2_ sterilization or sterile filtration of polymer precursors prior to gelation, as these methods better preserve the native structural hierarchy and biochemical cues essential for regenerative signaling [319]. Moist heat sterilization, such as autoclaving, denatures proteins like collagen, resulting in gelatin with altered bioactivity and mechanics. It is often undesirable for protein-based scaffolds designed to maintain natural structures, although it may be acceptable for silk fibroin or cellulose under certain conditions [319].

It is important to note that mechanotransduction guidelines developed for 2D surfaces do not necessarily apply linearly to 3D scaffolds. In 3D scaffolds, a high crosslinking density can limit cell spreading due to steric hindrance (small pore size), inhibiting the development of stress fibers even when the material is rigid. This might result in poor YAP nuclear localization despite high bulk stiffness, unless the matrix is degradable (for example, by MMPs), allowing cell dispersion. Thus, for 3D natural polymer scaffolds, degradability serves as a “permissive” gatekeeper for mechanotransduction [233].

**Figure 4:**
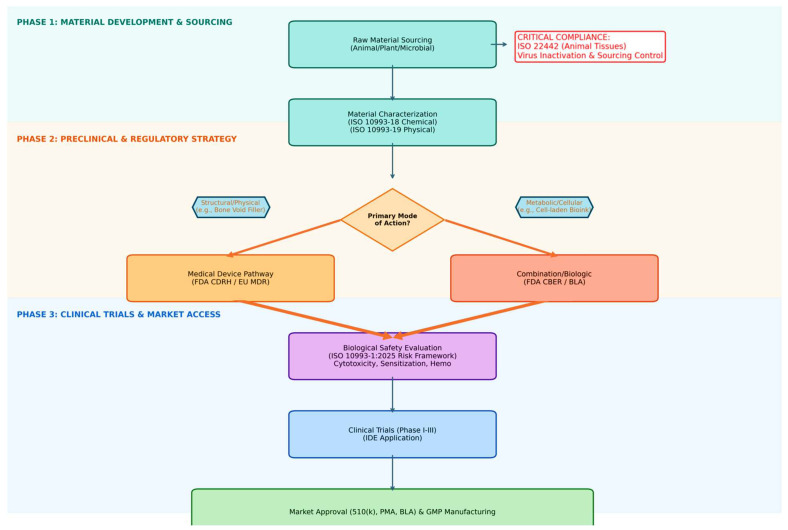
Flowchart of the development phases and steps: from raw material sourcing to market approval [325,326,327,328].

The scalability of composite formulations that combine polymers, mineral fillers, and bioactive molecules in accordance with good manufacturing practice (GMP) standards is still being developed (Figure 4). It is even harder to compare studies because there are no standard testing protocols, and the reported storage moduli, compressive strengths, degradation rates, and in vivo outcomes are all very different. Consensus reviews are increasingly stressing the need for consistent mechanical characterization, degradation profiling, and reporting of functional outcomes like bone volume fraction, cartilage integration scores, and electrophysiological recovery in nerve models. Setting up these kinds of standard frameworks would make it much easier for regulators to evaluate injectable scaffolds and would facilitate their clinical use [320,321,322,323,324].

Natural polymers show considerable batch-to-batch variation due to source heterogeneity and extraction techniques, in contrast to synthetic polymers with clearly defined molecular weights and purity profiles. Complying with ISO 10993 [329] biocompatibility standards and FDA requirements for repeatable manufacturing under GMP is made more difficult by this variability. Products based on decellularized ECM or natural polymers are often classified as medical devices (Class II or III) or combination products (biologic + device). The regulatory pathway (e.g., 510(k) vs. PMA in the US) depends heavily on whether the polymer is considered a structural component (device) or a drug delivery vehicle/metabolic agent (drug/biologic). Proving that the decellularization process effectively removes all DNA (typically <50 ng/mg dry weight) and cellular debris is a critical quality control metric to ensure immunocompatibility [330].

The clinical viability of naturally derived injectable hydrogels is demonstrated by an array of FDA- and EMA-approved products currently utilized in regenerative medicine and surgery. HA represents the most successful class of injectable natural polymers, with systems such as Synvisc-One^®^ (Hylan G-F 20) and Durolane^®^ widely employed for viscosupplementation in osteoarthritic joints. Beyond joint health, HA-based injectables like Restylane^®^ and Juvederm^®^ have defined the standard for soft tissue augmentation and dermal remodeling.

In the realm of tissue sealing and hemostasis, fibrin-based hydrogels such as Tisseel and Evicel^®^ are used globally as “biological glues” to facilitate tissue adherence and support the natural healing cascade post-surgery. Collagen-based injectables (e.g., Zyderm^®^) were among the first to be approved for soft tissue repair, though they have largely been superseded by HA due to lower immunogenicity. While many of these systems are primarily marketed for their mechanical or filler properties, they serve as the regulatory and technical blueprints for the next generation of “bio-instructive” hydrogels designed to deliver growth factors and stem cells directly to injury sites.

Validated assays for immune response characterization, in vivo degradation kinetics, and measurable functional outcomes like cardiac ejection fraction, nerve conduction velocity, cartilage integration scores, and bone volume fraction (BV/TV) are also necessary for clinical translation.

## 5. Conclusions

Naturally derived polymers present a promising approach for regenerative medicine, integrating biocompatibility, bioactivity, and customizable degradation with rapidly evolving manufacturing techniques. Quantitative evidence underscores their transformative potential: mineral-reinforced natural polymers facilitate up to 65% new bone formation in calvarial defects, nano-composite hydrogels enhance compressive performance near to that of native cartilage, and aligned hydrogels restore conduction velocities in peripheral nerve repair comparable to autografts. In cardiac applications, polymeric matrices facilitate functional recovery with over 50% increases in regional strain, whereas HA-based injectable systems decrease chronic ulcer healing time by as much as 40%. Such performance highlights significant clinical advantages beyond structural support, including the enhancement of osteogenesis, chondrogenesis, axonal extension, neovascularization, and inflammatory modulation.

Technological sophistication is also improving quickly: electrospinning is used to make ECM-like guidance cues, 3D bioprinting lets cells and growth factors mix in space, and 4D-printed structures let you change them dynamically and put them in place with little effort. Still, there are still regulatory and translational problems, such as changes in property caused by sterilization, differences between biological sources, and the need for standardized outcome reporting that connects mechanical metrics with biological function. For widespread clinical use, polymer chemistry, composite reinforcement, and scalable biomanufacturing researchers will need to keep coming up with new ideas. These materials are ready to change the way we do things by moving from passive implantation to active, biologically integrated regeneration in orthopedic, neurological, cardiovascular, and wound care settings.

## 6. Future Directions

Future investigations into natural polymer-based scaffolds should focus on developing adaptive, biologically responsive materials that can dynamically engage with their surrounding environment. Scaffolds could be engineered to self-heal following mechanical deformation and alleviate stress in a manner that facilitates cell spreading and migration by utilizing natural polymers functionalized with reversible chemistries, including boronate esters, Schiff bases, and host–guest interactions. Furthermore, enzyme-cleavable peptide crosslinkers, such as MMP-sensitive sequences, present an alternative approach to scaffolds that degrade only upon cellular remodeling initiation, thereby enabling constructs to mature concurrently with regenerating tissue. These materials, when combined with reinforcing phases like nanocellulose fibrils, chitin whiskers, or bioactive glass nanoparticles, could potentially provide the essential combination of ECM-like bioactivity and mechanical stability necessary for applications in cartilage, tendon, and bone regeneration.

Breakthroughs in fabrication, particularly in multi-material 3D and 4D printing, will enhance the possibilities of natural polymer composites. High-resolution bioprinting with modular bioinks (e.g., GelMA for cell adhesion, alginate for structural integrity, and hyaluronan for viscoelasticity) allows spatial patterning of biochemical and mechanical cues throughout a single construct. Integrating melt electrowriting or coaxial electrospinning can result in aligned microfibers for nerve-guiding or load-distributing lattices for osteochondral surfaces. Meanwhile, 4D printing techniques that use thermosensitive polymers (such as PNIPAAm-modified gelatin) or enzymatically responsive networks may result in implants that expand after insertion, reveal angiogenic peptides in response to hypoxia, or progressively change stiffness as tissues grow. These technologies enable the delivery of minimally invasive structures that automatically shift into their functional configurations within the body.

Injectable hydrogels are a third area where natural polymers can help with clinical translation, particularly those engineered with shear-thinning, self-healing, and stimuli-responsive properties. Hydrogels made of gelatin, chitosan, or modified hyaluronic acid can be designed to experience reversible network disruption upon injection yet restore mechanical strength shortly afterwards. Embedding microgels containing VEGF or SDF-1 can stimulate angiogenesis, whilst adding drug-loaded nanoparticles or cell-laden spheroids enables localized, long-term therapeutic administration. Such methods show great promise for treating irregular cartilage deformities, cardiac infarctions, and craniofacial applications that need minimally invasive administration. As these platforms become more integrated with computational design, patient-specific imaging, and GMP-compliant manufacturing, natural polymer-based materials will transition from passive scaffolds to intelligent, customizable biofabrication solutions that actively guide complex tissue regeneration.

## Data Availability

No new data were created or analyzed in this study.
